# Electro-Optic Kerr Response in Optically Isotropic Liquid Crystal Phases

**DOI:** 10.3390/ma17194926

**Published:** 2024-10-09

**Authors:** Tetiana Yevchenko, Dorota Dardas, Natalia Bielejewska, Arkadiusz C. Brańka

**Affiliations:** Institute of Molecular Physics, Polish Academy of Sciences, M. Smoluchowskiego 17, 60-179 Poznań, Poland; yevchenko@ifmpan.poznan.pl (T.Y.); bielejewska@ifmpan.poznan.pl (N.B.)

**Keywords:** Kerr constant, blue phases, liquid crystal mixture

## Abstract

The results of an experimental investigation of the temperature and wavelength dependence of the Kerr constant (*K*) of mixtures with an increasing amount of chiral dopant in an isotropic liquid crystal phase are reported. The material was composed of a nematic liquid crystal (5CB) and a chiral dopant (CE2), which formed non-polymer-stabilized liquid crystalline blue phases with an exceptionally large value of *K*∼2 × 10^−9^ mV^−2^. The measurements were performed on liquid and blue phases at several concentrations covering a range of temperatures and using three wavelengths: 532 nm, 589 nm and 633 nm. The work focused on changes caused by concentration and their impact on the increase in the value of *K*, and it was found that in the case of the 5CB/CE2 mixture these changes were significant and quite systematic with temperature and wavelength. It is shown that the dispersion relation based on the single-band birefringence model described *K* well in isotropic liquid crystal phases at all of the measured concentrations. In an isotropic fluid, both temperature-dependent parameters in the dispersion relation had a simple linear form and, therefore, the *K*-surface could be described by only four constants. In the blue phase, the expression reproducing the temperature variation of *K* depended on concentration, which could vary from being almost linear to quasi-linear and could be represented well by an inverse exponential analytic expression.

## 1. Introduction

In an optically isotropic medium, such as liquid dielectrics, isotropic liquid crystal (LC) mixtures, liquid crystal blue phases (BPs) or crystals with centro-symmetric point groups, the refractive indices are the same in all directions. Under the influence of an electric field, such a medium becomes optically anisotropic or birefringent with respect to the the optical axis parallel to the electric field direction, which means that the parallel and perpendicular refractive indices become unequal. Such behavior is due to a quadratic electro-optical phenomenon known as the Kerr effect, in which the induced birefringence, $\Delta n_{ind}$, is proportional to the square of the intensity of the externally applied electric field *E*:

(1) $$\Delta n_{ind}=\lambda KE^{2},$$

where *K* is the Kerr constant and λ is the wavelength of the incident light. In general, the Kerr effect is almost undetectable because it is much weaker than the effects proportional to *E*, but in an isotropic medium the first-order electro-optical effects disappear by symmetry and the Kerr effect becomes dominant. Consequently, the Kerr effect may provide a convenient electrically driven mechanism for switching between optically isotropic and anisotropic states, a process which is reversible. This general physical mechanism is exploited in liquid crystal blue phases (BPs), to modify their electro-optical properties and create the potential for applications in electro-optical devices [1,2,3].

In BPs, the liquid crystal molecules form a double-twisted helix or cylinder that self-assembles in a 3D amorphous or a cubic structure. Three types of BP are known: amorphous BPIII, a simple cubic BP (BPII) and a body-centered-cubic BP (BPI) symmetry structure, in order of increasing temperature [4,5,6]. In the cubic structures, the cylinders are separated by defect regions with a periodic spacing of several hundred nanometers [7,8]. Thus, blue phases form frustrated structures with crystal symmetry. They are formed typically in a narrow temperature range (1–2 °C) between the isotropic liquid phase and the chiral nematic (N*), which currently limits their utility in electro-optical devices.

To overcome this limitation, structural modifications—such as, e.g., polymer- stabilization [9,10], the use of nanoparticles [11,12] or unconventional molecules [13,14]—have been made, which have extended the BP’s temperature stability range considerably in certain systems. For example, stabilization with polymers may extend the temperature range even up to 60 °C and the use of nanoparticles or unconventional molecules up to 20 °C. Research into the blue phase is attracting a lot of interest, in part because of possible applications in next-generation displays and advanced optical devices, in which the Kerr effect constitutes the operation mechanism [15,16,17].

There are at least three main application advantages in using BP liquid crystals in electro-optical devices. First, they are optically isotropic, which reduces problems in aligning or orientating the working material in an optimum way. Secondly, the Kerr effect is fast, and it is possible to achieve submillisecond switching times [18]. Finally, BPs can exhibit a relatively large Kerr constant [1,19,20] (even up to two-orders-of-magnitude higher than that of nitrobenzene [21]), which allows for achieving a driving voltage value of order 10–20 V [22], a key practical advantage. Because of these potential practical advantages, the Kerr constant is a property of much interest in the liquid crystal field. Further progress largely relies on having a better understanding of how, for example, temperature (*T*), light wavelength (λ) and material composition affect the value of *K*. Determining the surface dependence K(T,λ) for any isotropic mixture of liquid crystals remains a difficult and basically unsolved task. Nevertheless, some partial descriptions or approximate formulas for this combined parameter dependence have been proposed. In the case of BPs, for example, a well-known phenomenological formula for the Kerr constant is the relation proposed by Gerber [23]. However, the Gerber formula exploits the properties of other liquid crystal phases, e.g., cholesteric phase, rather than the BP phase itself and does not provide an adequate means to study changes in the *K* of BP systems.

For many liquid crystal types there is a well-known linear relationship between temperature and 1/K in the liquid state that is obeyed by many materials. Furthermore, a consensus exists that this relationship is described well by the Landau–de Gennes theory [8,24], except perhaps in the temperature region close to the clearing temperature, $T_{c}$, which is defined as the transition temperature of the LC to its isotropic liquid phase. The form of K(T), in the case of the BPs, is less well-known. There is, however, some evidence that the Kerr constant in BPs can vary approximately linearly with the temperature [25,26,27,28]. Also, the λ dependence or dispersion of *K* has not been widely studied—in particular, in the case of BP materials. In liquid crystals, the birefringence dispersion follows from the existence of electronic absorption or molecular vibrational bands and must be taken into account. This issue was undertaken by Wu in a seminal paper on a single-band model [29], which, subsequently, was also generalized to the three-band model [30,31]. A more rigorous formulation of the dispersion equation based on the Vuks relation was proposed by Abdulhalim [32,33].

In our recent publication [28], hereinafter referred to as P1, a K(T,λ) surface relationship was proposed as a combination of the extended Kerr effect model [34,35] and the general single-band method by Abdulhalim [32]. The formula consists of an explicit λ dependence and two temperature-dependent parameters, denoted here by A(T) and B(T).

In this work, research on the K(T,λ) relationship is extended, mainly by examining the changes induced by increasing the amount of the chiral dopant in the LC mixture. Thus, a third main factor (i.e., the material composition) is also explored, which has a substantial influence on the value of *K*. In particular, with these mixtures, the scope of the applicability of the general description of the K(T,λ) surface in isotropic fluids is investigated. Also, the mixture 5CB/CE2 being a nonpolymerized system exhibits a very high Kerr constant [26], and how this unusually large value emerges from the doping by 5CB is not obvious. The microscopic origins of this large value of K, which is about two-orders-of-magnitude higher than the standard of nitrobenzene with $K∼4.5\times 10^{-12}$ mV^−2^, is not clear, but it is attributed to the dielectric anisotropy and birefringence in the underlying liquid crystal phase [26].

The work is organized as follows: in Section 2, the materials employed in the study and the experimental details are outlined; in Section 3, the formula for the K(T,λ)-surface used herein is discussed; the results are presented and discussed in Section 4; the main conclusions are summarized in Section 5.

## 2. Materials and Experimental Details

The experimental method for determining the Kerr constant applied in this work was described in detail in paper P1. In the method, the basis is the relationship between the induced birefringence and the intensity of the light, which is modulated by the electric field in the sample. The measurement of the second harmonic component of the intensity of the transmitted modulated light is performed using a modified in-plane switching cell geometry. The advantage of the method is that it uses a sufficiently small control voltage and can measure relatively small values of *K*, and it allows *K* to be measured directly, using two measurement voltages. It is worth noting that this method allows the texture of the sample to be previewed during the measurement, monitoring phase changes and sample alignment.

**Figure 1:**
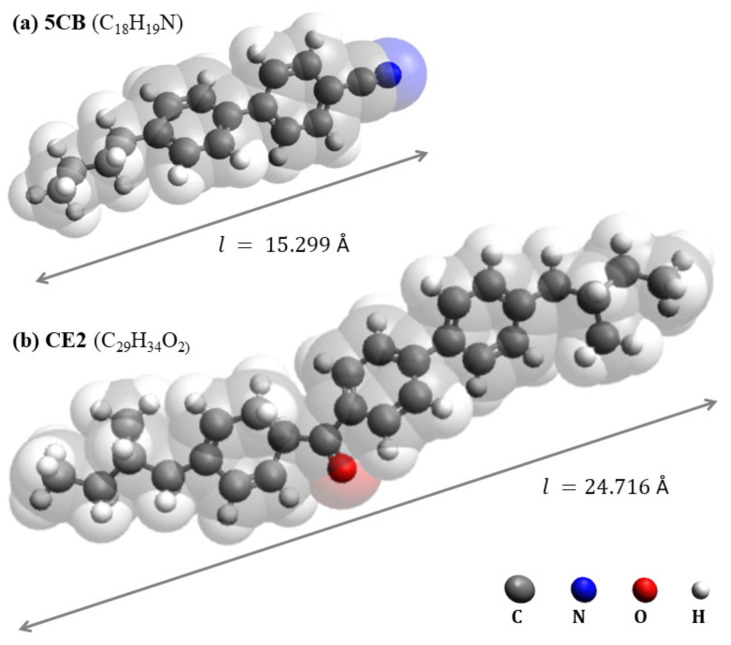
Chemical structure of the molecules forming the LC mixture used in this study: nematic host 5CB (**a**) and chiral dopant CE2 (**b**), where l is the length of the molecule. Elements are marked with colors: gray—carbon, blue—nitrogen, red—oxygen, white—hydrogen.

The materials used in this study were mixtures of the nematic liquid crystal 5CB (4-cyano-4′-pentylbiphenyl) with a chiral dopant CE2 [4″-(2-methylbutylphenyl)-4′-(2-methylbutyl)-4-biphenylcarboxylate (Merck KGaA, Darmstadt, Germany)], and they are shown in Figure 1. These mixtures were selected because they may form BPs and may exhibit a large Kerr constant compared to the values reported for other non-polymer-stabilized LC systems.

The measurement procedure details were similar to those given in P1. A sample was placed in the modified in-plane switching (IPSm) cell, which was mounted on a heater with a proportional–integral–derivative controller, PID UNIPAN type 650 (Scientific Instruments, Warsaw, Poland). The temperature was stabilized to within ±0.05 °C. A replaceable light-emitting diode (LED) was used as the light source. Additionally, to obtain essentially a monochromatic light source a suitable filter was placed after the diode. The dispersion effect was studied by measurements at three light wavelengths: $\lambda_{1}=532$ nm, $\lambda_{2}=589$ nm and $\lambda_{3}=633$ nm. An alternating current (AC) signal of 1 kHz, generated by a DS340 (Stanford Research Systems Inc., Sunnyvale, CA, USA) function generator with a wideband linear amplifier F20A (FLC Electronics, Partille, Sweden), was applied to the sample. In these measurements, the rms value of the supplied alternating voltage UAC was less than 100 V. The light intensity was detected by a Si PiN (FEMTO Messtechnik GmbH, Berlin, Germany) photodiode and transmitted to a digital multimeter (34401A Hewlett Packard, CA, USA) and an SR530 phase-sensitive amplifier (Stanford Research Systems, Sunnyvale, CA, USA). The signal components, DC and second harmonic, were measured in parallel, using a multimeter and a lock-in amplifier, respectively. The cells (IPSm) used for the measurements were self-made from two cut-glass plates. Two copper electrodes of thickness *d* were placed between them, separated by a distance *l*. In this work, the results concerned the cases when d=20 μm and *l* = 500–700 μm, which was considered optimal for these measurements, according to previous studies [28]. The quality of the measuring equipment and the sample cell gave values of the Kerr constant with uncertainties up to 10%.

## 3. K(T,λ) Surface

As was mentioned in the Introduction, *K* depends implicitly on different factors, such as the light wavelength, temperature and material composition, and its comprehensive description over sufficient parameter ranges is not yet available. For example, the dispersion of *K* for LC materials and especially for BPs is hardly ever reported, even though it is quite relevant in designing devices using liquid crystals.

In order to establish K(T,λ) for optically isotropic LC, a combination of a single-band birefringence dispersion model [29,32,33] and the saturated birefringence has been proposed [28,36] recently. In this approach, the phenomenological extended Kerr effect model [34,35] is exploited, which calculates the induced birefringence:

(2) $$\Delta n_{\mathrm{ind}}=\Delta n_{s}\left[1-\exp\left(-{\left(\frac{E}{E_{s}}\right)}^{2}\right)\right],$$

where $E_{s}$ and $\Delta n_{s}$ represent the saturation electric field and the saturated induced birefringence, respectively. For the low electric field $E/E_{s}\ll 1$, this extended Kerr effect reduces to the Kerr effect in Equation (1), and

(3) $$K=\frac{\Delta n_{s}}{\lambda E_{s}^{2}}.$$

Next, the single-band birefringence dispersion model [29] based on the Vuks relation is applied, obtaining $\Delta n_{s}=G\lambda^{2}\lambda^{*2}/\left(\lambda^{2}-\lambda^{*2}\right)$, where *G* is a constant and $\lambda^{*}$ stands for the wavelength of the nearest absorption band of the liquid crystal material [29]. In the result, via Equation (3), the following dispersion relation is proposed [36]:

(4) $$K=\frac{A\lambda }{\lambda^{2}-B},$$

where $A=\lambda^{*2}G/E_{s}^{2}$ and $B=\lambda^{*2}$. A slightly more general model, in which the sensitivity to wavelength in the Vuks equation is taken [32,33], is $\Delta n_{s}=\left[\sqrt{G_{1}\lambda^{2}-1}-\sqrt{G_{2}\lambda^{2}-1}\right]/\sqrt{G_{3}\lambda^{2}-1}$, where $G_{1},G_{2},G_{3}$ are parameters that depend on the temperature and the LC material parameters. This model for $\Delta n_{s}$ is a good approximation for the relation in Equation (4), in which $B=1/2G_{3}$ and $A=\left[\sqrt{G_{1}}-\sqrt{G_{2})}\right]/2\sqrt{G_{3}}E_{s}^{2}$ are the temperature- and material-dependent parameters [28]. Below, in Section 4, the formula in Equation (4) with A(T), B(T) is used to represent the K(T,λ) experimental data for the 5CB/CE2 investigated mixtures.

## 4. Results and Discussion

From a direct observation of the polarizing optical microscope textures and application of the image analysis method [37], the phase sequences of the mixtures were determined, which are given in Table 1. The $T_{c}$ values were also confirmed, using the DSC (differential scanning calorimetry) thermograms. In what follows, the pure 5CB was considered as a zero percent mixture that enabled the changes caused by the chiral dopant and temperature on the phase sequences to be compared. At low concentrations of up to 20 wt.% of chiral dopant, no BP was formed. At 25 wt.% of chiral dopant, only the BPII phase appeared. For higher concentrations, two blue phases (BPI and BPII), were observed. As the concentration increased further, the range of BP formation increasingly reduced, and for concentrations above 50 wt.% of chiral dopant they were hardly detectable. Therefore, the addition of a chiral dopant may significantly affect the properties of BPs and what can be used as a mechanism for changing the value of *K*.

**Figure 2:**
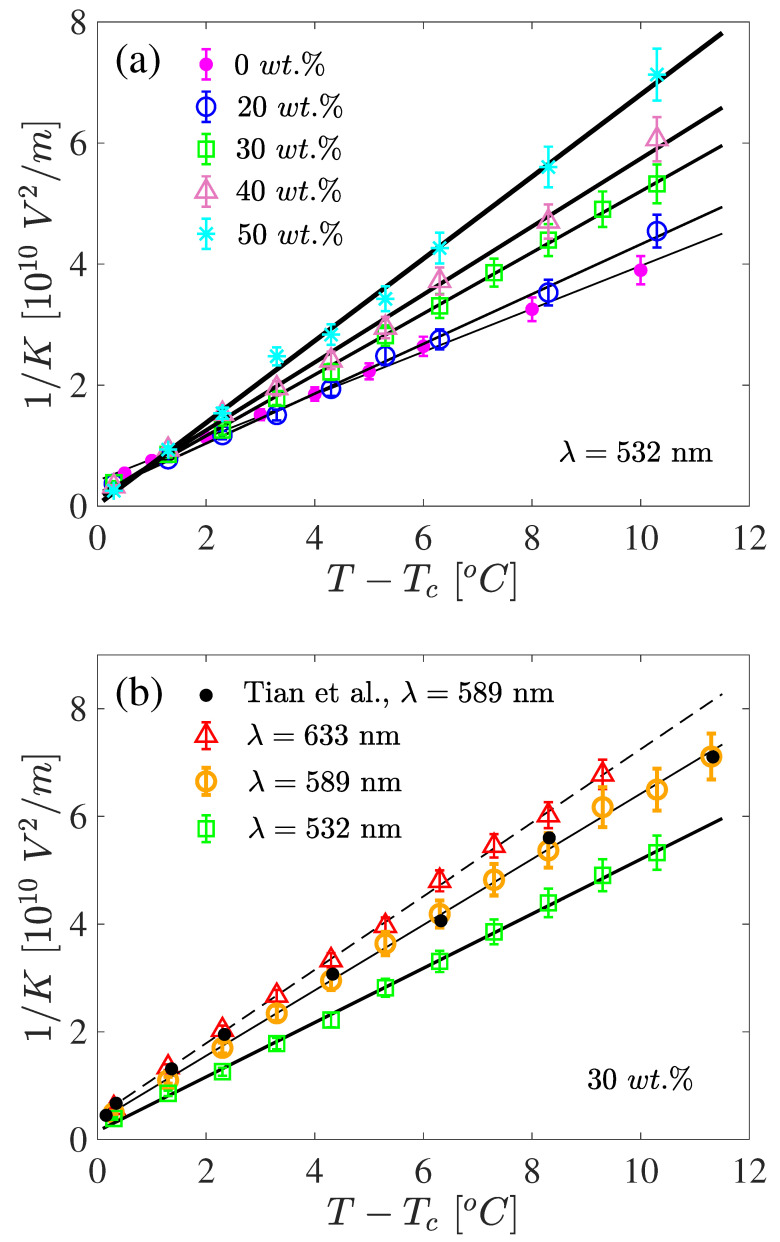
The inverse of the Kerr constant of the 5CB/CE2 mixtures as a function of the temperature above the clearing temperature, $T_{c}$. In panel (**a**), the results are for one wavelength (λ=532 nm) and different concentrations given in the graph. In panel (**b**), the results are for one concentration (30 wt.%) and three wavelengths. The symbols are the data of this work and the black filled-in circles in (**b**) are from [26]. The lines are linear fits to the experimental data of this work.

The Kerr constant in the liquid phase, i.e., above the clearing temperature, is shown in Figure 2. In the figure, the concentration (Figure 2a) and wavelength (Figure 2b) dependence is illustrated. As may be seen in the figure, the linear temperature dependence of 1/K,

(5) $$\frac{1}{K}=\frac{T-T^{*}}{C}=\frac{\Delta T-\Delta T^{*}}{C},$$

was obeyed well. The same linear behavior was obtained for all mixtures and λ values considered in this work. In the above relation, *C* is a constant and $T^{*}$ is the temperature at which 1/K extrapolates to zero and $\Delta T=T-T_{c}$, $\Delta T^{*}=T^{*}-T_{c}$. Such a simple temperature dependence is well-documented for many materials, at least for temperatures not too close to $T_{c}$, where deviations from the linear trend have been reported [38].

**Figure 3:**
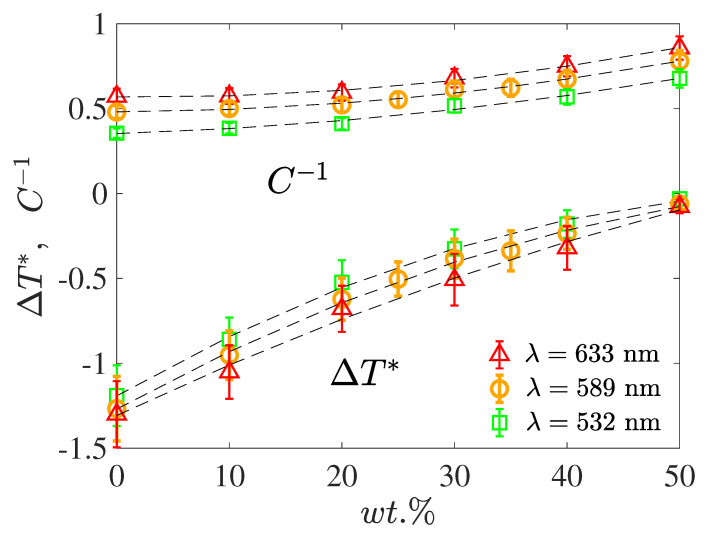
The least squares linear fit parameters of 1/K data, such as in Figure 2, of the 5CB/CE2 mixtures for three λ as a function of the CE2 dopant concentration. The symbols are the experimentally derived slope $C^{-1}$, and the $\Delta T^{*}$ values and the dashed lines are meant to guide the eye.

The presence of CE2 in 5CB caused a faster decrease of *K* (i.e., a slope of 1/K was larger) with temperature. Close to the clearing temperature, the lines crossed and, hence, the Kerr constant became higher than that of pure 5CB (see Figure 2a). Also, we observed the same trend, in that the more dopant in the mixture the more the $T^{*}$ value shifted towards $T_{c}$. Therefore, by changing the amount of chiral dopant, the value of *K* could be influenced significantly. For example, by lowering the concentration from 50 wt.% to 10 wt.% the *K* value almost doubled. It is worth adding that in the case of the mixtures considered this impact was quite systematic, but this may not be a common phenomenon for other LC systems. This is shown in Figure 3, where the constants $C^{-1}$ and $T^{*}$ are given for all measured concentrations and three different wavelengths. In Figure 3, the symbols are the parameter values taken from the straight line fits to the experimental data, such as those presented in Figure 2. As may be seen, both parameters $C^{-1}$ and $T^{*}$ increased monotonically with dopant concentration. The constant $T^{*}$ was lower than $T_{c}$ (i.e., $\Delta T^{*}<0$), in accordance with the Landau–de Gennes model [8,24], and at high concentration it reached virtually $T_{c}$, i.e., $\Delta T^{*}\to 0$. The wavelength differences or dispersion splitting of the slope remained practically the same for all concentrations. For $\Delta T^{*}$, the observed splitting was largely within the accuracy of the results, and it became very small at higher concentrations.

**Figure 4:**
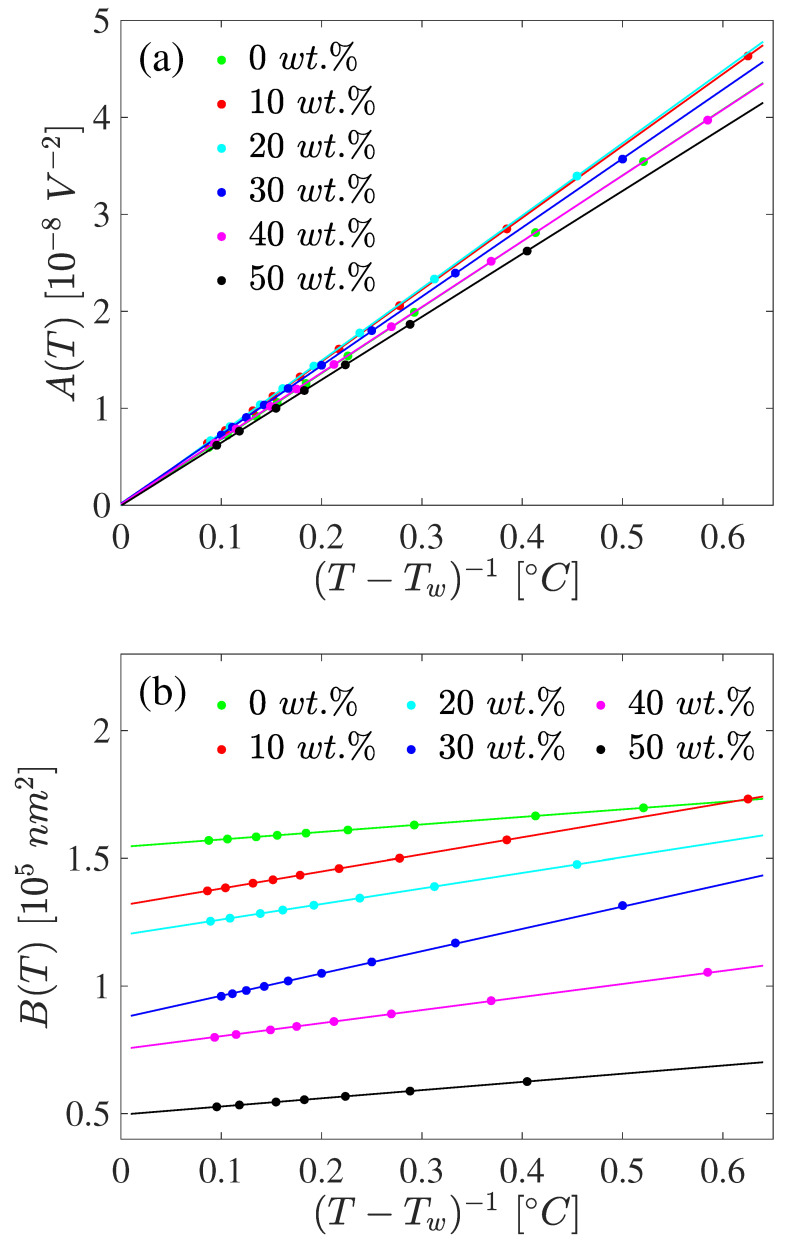
The variation of *A* (panel (**a**)) and *B* (panel (**b**)) defined in Equation (4) as a function of temperature ${\left(T-T_{w}\right)}^{-1}$ for the dopant concentrations given in the figure key. The solid circles are obtained from the data of Figure 3 for each concentration. The solid lines in panels (**a**,**b**) are linear regression fits with Equations (6) and (7), respectively.

Employing the scheme in Section 2, it was verified that the formula in Equation (4) was well obeyed for each temperature (at least for Δ*T* > 0.2 °C) and for all three wavelengths. From the results given in Figure 3 for different λ, the temperature dependence of *A* and *B* were obtained for each concentration, and they are shown in Figure 4. From this figure, it follows that for each mixture both A(T) and B(T) were linear in ${\left(T-T_{w}\right)}^{-1}$, where $T_{w}$ was a constant which, in general, was different from $T^{*}$. Also, in the limit $(T-T_{w})\to 0$ the *A*-function tended to zero. Thus, the Kerr constant obtained for the mixtures in their liquid phase is well-described by the relation in Equation (4), in which

(6) $$A\left(T\right)=\frac{a_{1}}{T-T_{w}}$$

and

(7) $$B\left(T\right)=\frac{b_{1}}{T-T_{w}}+b_{2}.$$

From these semi-empirical forms, the K(T,λ) surface of the 5CB/CE2 mixture could be estimated, using only the four constants $a_{1},b_{1},b_{2}$ and $T_{w}$. This is noteworthy, as it confirms the suggestion made in P1 that in isotropic liquids both parameters *A* and *B* in the dispersion relation given in Equation (4) can have a simple linear *T*-dependence. It is also clear from Figure 4 that a change in concentration of the chiral dopant influences considerably the values of the function *B*, while the function *A* is less sensitive to that change.

It is also worth noting that on inserting *A* and *B* from Equations (6) and (7) into the formula in Equation (4), the linear formula in Equation (5) can be recovered with $C=\lambda a_{1}/\left(\lambda^{2}-b_{2}\right)$ and $T^{*}=T_{w}+b_{1}/\left(\lambda^{2}-b_{2}\right)$. As far as we are aware, this is a new and more explicit λ-representation of the *C* and $T^{*}$ quantities. From these representations, it directly follows that there is a stronger dispersion effect on 1/C than for $T^{*}$. Thus, the linear form of *A* and *B* implies the linear temperature dependence of 1/K or, alternatively, the linear temperature dependence of 1/K may be viewed as a consequence of the linear form of the *A* and *B* functions.

**Figure 5:**
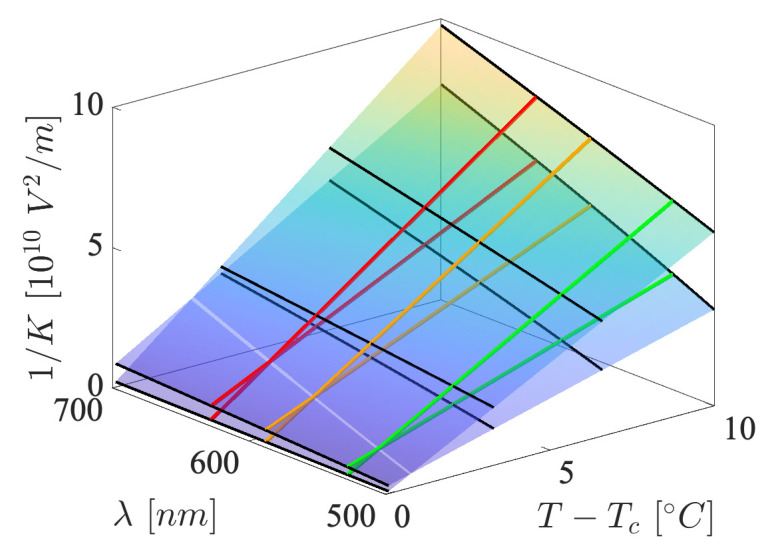
Temperature and wavelength (dispersion) dependence of the inverse Kerr constant of two 5CB/CE2 liquid mixtures, with dopant concentrations of 10 wt.% and 50 wt.%. The colored solid lines are 1/K(T) for three wavelengths: $\lambda_{1}$, $\lambda_{2}$ and $\lambda_{3}$. Examples of 1/K(λ) are denoted as solid black lines. The surface is given by the relations in Equations (4) and (7) with the parameters $T_{w}=-1.3$, $a_{1}=7.4146\cdot 10^{-8}$, $b_{1}=66,626$ and $b_{2}=131,570$ for concentration 10 wt.%, and $T_{w}=-0.17$, $a_{1}=6.4931\cdot 10^{-8}$, $b_{1}=32,102$ and $b_{2}=49,603$ for concentration 50 wt.%.

Examples of the 1/K(T,λ) surfaces of the 5CB/CE2 liquids are shown for two concentrations, 10 wt.% and 50 wt.%, in Figure 5. As may be seen, the surfaces were very flat, monotonically increased with *T* and λ, and crossed near $T_{c}$. An intersection line may be seen, which determined the regions in which the dopant lowered and increased the *K* value.

As mentioned at the beginning of this section, the influence of the CE2 dopant on the behavior of pure 5CB at lower temperatures (i.e., below the clearing point) was fairly significant. First of all, no BPs were formed between the Iso and N* phases for concentrations in the range ≤20 wt.%. For higher concentrations, the BPII was formed but its range decreased substantially at higher concentrations, making its detection largely intractable for >50 wt.%. Thus, as may be expected, the behavior of K(T,λ) in the BPII became more complex than in its isotropic liquid phase. Also, in general, the form of K(T) is much less well-known in BP phases than for isotropic fluids. It follows from our observations that for mixtures with higher CE2 concentrations the K(T,λ) surface is quite similar to that shown in Fig. 7 in P1, i.e., K(T) is only quasi-linear with a characteristic S-like form, and the *A* and *B* functions have no obvious simple *T*-dependence. In fact, different higher order polynomial fits are required for each concentration.

**Figure 6:**
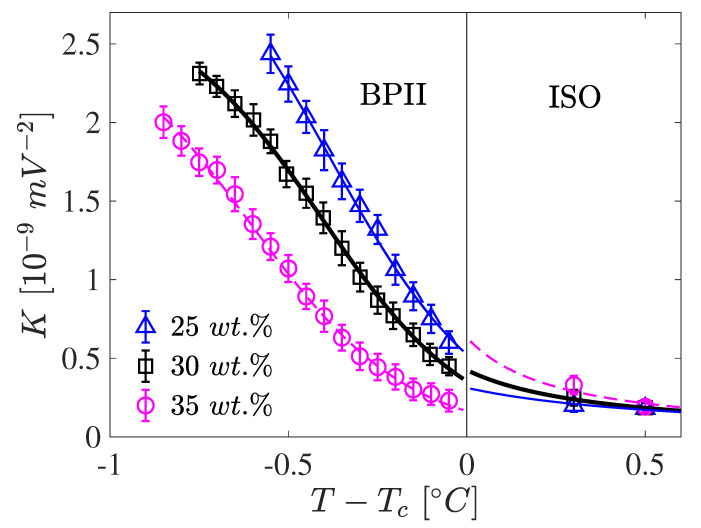
The Kerr constant as a function of temperature in the BPII phase and isotropic phase in the 5CB/CE2 mixtures for λ = 589 nm and three concentrations. In the BPII phase, the symbols are the experimental data and the solid line is the *K* representation given in Equation (8), with the constants ($W\times 10^{-9},D\times 10^{-9},\eta$) for concentrations 25 wt.%, 30 wt.%, 35 wt.%, respectively, being (0.280,1.6265,−4.5608), (0.359,2.4681,−4.7387), (0.370,−4.5362,5.7402). The constants *D*, *W* are in 1/K units, i.e., $V^{2}/m$ and η in °C^−1^.

However, some noticeable changes occurred in the concentration range at which BP began to form, i.e., about 25 wt.%. As shown in Figure 6, at this concentration the highest *K* values were achieved. Further increase of the concentration of CE2 caused a systematic lowering of *K* values in BPII. Nevertheless, their values were very high and, in comparison to those of the isotropic liquid (i.e., above the $T_{c}$), were at least an order of magnitude higher.

Probably the most noteworthy feature was the change in the analytic form of the K(T) function, which went from being an almost perfectly linear function at low concentration to changing gradually into a quasi-linear or S-like shape at higher concentrations. Our results suggest that, in general, for LC mixtures that form BP, depending on the concentration of the chiral additive used, the K(T) of the BP can exhibit both linear or non-linear characteristics. This partially explains the success in previous work of using a linear relationship for the K(T) function in the BP phases [25,27,39,40]. For the mixtures studied in this work, the linear temperature dependence appeared with the formation of the BP phase, and then, on increasing the CE2 concentration, an S-shape represented the K(T) better in this BPII phase. As shown in P1, the temperature dependence of *K* may be well described by the three-parameter formula:

(8) $$K≅\frac{1}{D\exp(-\eta \left(T-T_{c}\right))+W},$$

where *W*, η and *D* are constants.

It must be said that the formula in Equation (8) is not unique, and its general utility needs more experiments with other materials to discover whether it also applies to other LC systems.

The results obtained for the mixtures shown in this study indicate that K(T) can change discontinuously in the transition from the BPII to the isotropic liquid phase, especially if linear extrapolation (Equation (5)) in the fluid phase is used. Our results show that the size of the discontinuity can vary significantly and not in a systematic manner with the concentration change. As in the fluid for small $T-T_{c}$, some departure from the linear dependence of 1/K(T) is not excluded [38], and reduction or elimination of the discontinuity may also be possible, but further studies near the transition region are required, to address this issue more conclusively.

## 5. Conclusions

In this work a recently proposed experimental scheme devised by us for measuring the Kerr constant, *K*, in liquid crystals has been used to discover how the concentration of the chiral CE2 dopant in 5CB/CE2 mixtures changes the temperature and wavelength dependence of *K*. The mixtures form blue phases (BPs) and possess large values of *K*, which makes them a suitable liquid crystal material for such a study. The experimental measurements were performed on the liquid and BPII phases with several dopant concentrations, and for a range of temperatures at three wavelengths.

In general, changing the concentration of components in liquid crystal mixtures is the main way of controlling the mesophase structure and the derived material properties. Such an approach has often been used in the case of BPs, which are considered potential materials for electro-optical devices in which the Kerr effect is used as a physical mechanism for switching optical states. In this context, studies of the influence of various factors on the increase of *K* is highly topical and remains a largely unexplored area, mainly due to the potential sensitivity of the Kerr constant on *T* and λ, which is also a property that is difficult to measure experimentally, particularly for BPs.

The experimental measurements performed in this study have shown that the dispersion relation in Equation (4) describes the experimental data very well. Thus, the work provides further confirmation that this relation can describe well the wavelength dependence (dispersion) of the Kerr constant in different optically isotropic materials.

In the isotropic liquid phase in all the measured mixtures the temperature dependence followed well the simple linear relationship, 1/K∼T, and a change in the dopant concentration influenced the *K* value considerably. Basically, adding CE2 reduced *K*, and for the largest concentrations studied this reduction was quite substantial. For temperatures close to the clearing temperature, the inverse trend was observed, i.e., adding CE2 appeared to increase slightly *K*, but more detailed studies are needed to confirm or disprove this behavior and its possible general occurrence for other liquid crystal mixtures.

An important and potentially useful finding is that for 5CB/CE2 mixtures in an isotropic liquid the temperature dependence of the two functions *A* and *B* (defined in Equations (6) and (7)) in the dispersion relation have a simple linear form. This enables a description of the K(T,λ) surface, in terms of only four constants (numbers), to be formulated. The influence of the concentration on *K* is mainly reflected in the *B*-function. Also, the *A* and *B* linearity in temperature implies the commonly observed linear form of 1/K(T) for different isotropic liquids. The results obtained here suggest that this linear relationship may be a general feature of isotropic liquids, but further experiments would be required, to test this.

The transition from liquid to the BPII phase caused about an order-of-magnitude increase in the Kerr constant value. Its wavelength and temperature dependence was similar to that found in P1 for a dopant concentration of 30 wt.%. The influence of the concentration was a significant issue, and it was found here to follow the same trend as in the liquid phase—that is, increasing the amount of chiral dopant reduced the *K* value (the largest value was for the concentration initiating the formation of BPII). It was also found that for the lowest CE2 concentrations the character of K(T) was practically linear, and then, with increasing concentration, it gradually changed to quasi-linear, with clear departures from the linear trend. We consider that this is a noteworthy observation, as it demonstrates the non-triviality of the temperature dependence of the Kerr constant of a blue phase at high dopant concentrations. On the other hand, this experimental study confirms to a large extent the accuracy of a linear temperature dependence of the Kerr constant for certain BPs and dopant concentration ranges. Finally, it should be emphasized that the obtained trends of changes caused by concentration found in this study were very systematic and should be attributed to this particular mixture. Such a smooth or continuous variation in behavior and the exceptionally high *K* values make the 5CB/CE2 mixture a particularly valuable LC material for further studies of *K* and related properties. In such studies, the important role of the pi–pi stacking interactions in stabilizing the binding between 5CB molecules, demonstrated recently by Xia et al. [41], is worth taking into account. 

## Figures and Tables

**Table 1:** Phase transition temperatures for the different 5CB/CE2 mixtures. The concentration of dopant is given as a percentage by weight (wt.%).

Mixture [wt.%]	Phase Transitions [°C]
0	I(35.10)N
10	$I\;\left(41.80\right)\;N^{*}$
20	$I\;\left(49.40\right)\;N^{*}$
25	$I\;\left(52.20\right)\;\mathrm{BPII}\;\left(51.60\right)\;N^{*}$
30	$I\;\left(58.45\right)\;\mathrm{BPII}\;\left(57.69\right)\;\mathrm{BPI}\;\left(57.05\right)\;N^{*}$
35	$I\;\left(62.45\right)\;\mathrm{BPII}\;\left(61.60\right)\;\mathrm{BPI}\;\left(61.00\right)\;N^{*}$
40	$I\;\left(68.10\right)\;\mathrm{BPII}\;\left(67.60\right)\;\mathrm{BPI}\;\left(65.30\right)\;N^{*}$
50	$I\;\left(77.50\right)\;\mathrm{BPII}\;\left(77.10\right)\;\mathrm{BPI}\;\left(77.00\right)\;N^{*}$

## Data Availability

The raw data supporting the conclusions of this article will be made available by the authors on request.
